# Conditioned medium derived from 3D tooth germs: A novel cocktail for stem cell priming and early in vivo pulp regeneration

**DOI:** 10.1111/cpr.13129

**Published:** 2021-09-28

**Authors:** Tengfei Zhou, Mingdeng Rong, Zijie Wang, Hongxing Chu, Chuying Chen, Jiayi Zhang, Zhihui Tian

**Affiliations:** ^1^ Department of Periodontology and Oral Implantology Stomatological Hospital Southern Medical University Guangzhou China; ^2^ Department of Stomatology Nanfang Hospital Southern Medical University Guangzhou China; ^3^ School of Stomatology Southern Medical University Guangzhou China

**Keywords:** cell priming, conditioned medium, mesenchymal stem cells, regenerative medicine, secretome

## Abstract

**Objectives:**

Conditioned medium (CM) from 2D cell culture can mitigate the weakened regenerative capacity of the implanted stem cells. However, the capacity of 3D CM to prime dental pulp stem cells (DPSCs) for pulp regeneration and its protein profile are still elusive. We aim to investigate the protein profile of CM derived from 3D tooth germs, and to unveil its potential for DPSCs‐based pulp regeneration.

**Materials and Methods:**

We prepared CM of 3D ex vivo cultured tooth germ organs (3D TGO‐CM) and CM of 2D cultured tooth germ cells (2D TGC‐CM) and applied them to prime DPSCs. Influences on cell behaviours and protein profiles of CMs were compared. In vivo pulp regeneration of CMs‐primed DPSCs was explored using a tooth root fragment model on nude mice.

**Results:**

TGO‐CM enhanced DPSCs proliferation, migration, in vitro mineralization, odontogenic differentiation, and angiogenesis performances. The TGO‐CM group generated superior pulp structures, more odontogenic cells attachment, and enhanced vasculature at 4 weeks post‐surgery, compared with the TGC‐CM group. Secretome analysis revealed that TGO‐CM contained more odontogenic and angiogenic growth factors and fewer pro‐inflammatory cytokines. Mechanisms leading to the differential CM profiles may be attributed to the cytokine–cytokine receptor interaction and PI3K‐Akt signalling pathway.

**Conclusions:**

The unique secretome profile of 3D TGO‐CM made it a successful priming cocktail to enhance DPSCs‐based early pulp regeneration.

## Graphical abstract

The capacity of conditioned medium from 3D cell culture to prime dental pulp stem cells (DPSCs) and its protein profile remain elusive. Our results suggested that conditioned medium derived from 3D cultured tooth germ organs (3D TGO‐CM) can function as a priming cocktail to enhance DPSCs‐based early pulp regeneration due to unique protein profile.

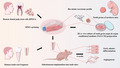

## 1 INTRODUCTION

Dental pulp stem cells (DPSCs) offer practical advantages such as accessibility, self‐renewal, multipotency, and immunomodulation,^1^ making DPSC‐based tissue engineering increasingly attractive in pulp–dentin complex regeneration from bench to bedside.^2^, ^3^ However, direct mesenchymal stem cells (MSC) administration presents limitations such as transplanted cells' low survival, trans‐differentiation rates,^4^ and compromised regenerative functions.^5^ Donor systemic diseases and senescence can also impair the MSC reparative capacity,^6^, ^7^ with unsatisfactory clinical trial outcomes.^8^, ^9^, ^10^

In vitro MSC priming is a promising technique in circumventing these shortcomings by modulating the secretome,^11^ with hypoxia, cytokines, and 3D cell culture proposed as in vitro priming stimuli.^12^, ^13^, ^14^, ^15^ Conditioned medium (CM), the supernatant collected from the cell culture medium, contains the MSC secretome, which includes extracellular matrix components, growth factors, and cytokines.^16^ Already being broadly used,^17^ CM priming can promote tissue regeneration, angiogenesis, immunomodulation, and anti‐fibrosis.^18^ DPSC‐derived CMs can recapitulate parent MSC functions, suggesting potential in dental and extra‐oral tissue engineering applications.^19^

Most current CM types are harvested from 2D cell culture,^17^ including tooth germ cell‐derived CM (TGC‐CM).^20^, ^21^, ^22^ Nevertheless, living systems like tooth germ organs (TGOs) exist in well‐organized 3D arrangements with intricate cellular and extracellular interactions; these in vivo bioprocesses can barely be replicated by 2D monolayer cell cultures. In contrast, 3D cell cultures create more accurate physiological simulations of in vivo microenvironments, with gene and protein expression closely resembling those within original living systems.^23^, ^24^ As pulpogenesis is orchestrated by bio‐factors secreted by mesenchymal and epithelial cells within the tooth germ during embryonic development,^25^ we reasonably deduced that tooth germ‐derived CM would contain a semblable tooth germ protein profile which could orchestrate pulp repair under physiological and pathological scenarios.

While the literature on 2D CM‐based DPSC‐priming abounds, few studies have addressed 3D CM‐based DPSC‐priming for pulp regeneration.^19^ Only a few studies have analysed the protein profile of 2D CM derived from DPSCs.^26^, ^27^ It remains unclear whether and how CM of 3D tooth germ organ (TGO‐CM) will impact on pulp regeneration.

Due to its secretome biomimicry, we hypothesized that 3D TGO‐CM would triumph over its 2D counterpart in promoting early pulp regeneration. We compared the effects of 3D TGO‐CM and 2D TGC‐CM on in vitro DPSC proliferation, migration, differentiation, and mineralization, and on in vivo pulp regeneration after 4 weeks. Additionally, we analysed the compositional differences between the two CMs and explored the resulting biomechanisms. To the best of our knowledge, this study is the first to report the protein profile of 3D cultured tooth germs and the first to unveil its potential in priming DPSCs for early in vivo pulp regeneration. Our results may shed light on stem cell priming for pulp regeneration using a trace‐back‐to‐organ approach.

## 2 MATERIALS AND METHODS

### 2.1 Cell culture and CM preparation

#### 2.1.1 Isolation and cell culture of human DPSCs and mouse TGCs

Newly extracted healthy human third molars and premolars were collected from healthy anonymous patients (18–25 years), with approval from the Institutional Review Board of the Stomatological Hospital, Southern Medical University (201806), and informed consents from all patients. Teeth were stored in culture medium and immediately delivered to the laboratory for further processing. Mandibular first molar tooth germs from newborn CD‐1 mice were surgically dissected following a previously reported protocol.^28^ All animal experiments conformed to related ethical principles.

Human DPSCs and mouse TGCs were isolated as previously reported^28^ and cultured in L‐DMEM supplemented with 15% foetal bovine serum (FBS), 100 U/ml penicillin‐G, and 100 mg/ml streptomycin, under 5% CO_2_ at 37°C. The culture medium was refreshed every 2–3 days. Cells were passaged when they reached 80% confluency and expanded for all experiments at passages 3–5.

#### 2.1.2 Immunofluorescent staining of mouse TGCs

Immunofluorescent staining was performed as per the manufacturer's instructions, using anti‐CK14 and anti‐vimentin antibodies (Abcam, Cambridge, UK) and treated with related secondary antibodies. Samples were blocked using Vectashield mounting medium containing DAPI. Images were captured using a confocal immunofluorescence microscope.^29^

#### 2.1.3 Preparation of 2D TGC‐CM

At 80% confluency, the TGC medium was replaced with FBS‐free L‐DMEM. After 16 h, the resulting medium was collected, filtered, and centrifuged at 1,000 rpm for 5 min at 4°C. The supernatant was harvested as 2D TGC‐CM, stored at −80°C, and used without concentration.

#### 2.1.4 Preparation of 3D TGO‐CM

Fifteen TGOs of newborn mice were 3D ex vivo cultured in 1‐ml FBS‐free L‐DMEM under 5% CO2 at 37°C. The medium was refreshed at the same time intervals as the TGC‐CM culture. At 8, 16, and 24 h, the resulting medium was collected, filtered, and centrifuged at 1,000 rpm for 5 min at 4°C. The supernatant was harvested as 3D TGO‐CM, stored at −80°C, and used without concentration.

#### 2.1.5 Hoechst/TUNEL staining of TGOs

Paraffin‐embedded TGO samples were sectioned into 5‐μm slices at timed intervals, stained using a TUNEL Apoptosis Detection Kit (Vazyme Biotech, Nanjing, China) following the manufacturer's protocol, and subjected to Hoechst 33342 staining (Solarbio, Beijing, China). Unstained sections were stained with hematoxylin and eosin (H&E; Solarbio). The images were visualized using a fluorescent microscope.

#### 2.1.6 Cell viability assay of TGOs

A Live Dead Cell Viability Assay Kit (Sigma‐Aldrich, St. Louis, MO) was used according to the manufacturer's protocol. TGOs at 8, 16, and 24 h were stained with calcein AMAM (green, live cells) and propidium iodide (red, dead cells). Images were captured using a fluorescence microscope.

### 2.2 In vitro DPSCs behaviours

#### 2.2.1 DPSCs priming with CMs

Conditioned mediums harvested at 16 h were used to prime DPSCs. L‐DMEM supplemented with 15% FBS was used in the control medium group in all assays. In the TGC‐CM and TGO‐CM group, L‐DMEM was supplemented with 15% FBS for cell proliferation and migration assays or supplemented with 15% FBS containing related osteoinductive ingredients (1% penicillin/streptomycin, 50 μg/ml ascorbic acid, 10 nmol/L dexamethasones, and 5 mmol/L β‐glycerophosphate) for in vitro odontogenic/osteogenic differentiation assays. All groups were incubated under 5% CO_2_ at 37℃.

#### 2.2.2 Cell proliferation assay

Dental pulp stem cells were seeded at 10,000/well in 96‐well plates and cultured for 5 days. CCK‐8 assay was conducted as per the manufacturer's protocol on days 1–5, and cell viability was measured via UV spectrophotometry using absorbance at 450 nm.

#### 2.2.3 Transwell cell migration assay

Assays were conducted, according to the manufacturer's instructions. In brief, DPSCs cultured in TGC‐CM, TGO‐CM, and control medium were digested and resuspended, respectively. 2 × 10^5^ DPSCs from each of three groups were seeded into the upper chambers containing FBS‐free L‐DMEM in all groups. The lower chambers contained 15% FBS‐supplemented L‐DMEM in all groups. After a 24‐h incubation, migrated cells were stained with 0.1% crystal violet, and quantitative analysis was performed by cell counting using ImageJ software (National Institute of Health).

### 2.3 In vitro odontogenic differentiation

#### 2.3.1 Alizarin Red S staining

Osteogenic inductive ingredients were added into all medium groups when DPSCs reached 80% confluency. After 3 weeks, cells were fixed with 4% paraformaldehyde, washed, and stained with Alizarin Red S (Sigma‐Aldrich) for 20 min. The formation of mineralized nodules was observed under a microscope. Quantitative data were obtained by solubilizing the samples with 10% cetylpyridinium chloride solution (CPC, Sigma‐Aldrich) and measuring their optical density at 490 nm.

#### 2.3.2 Quantitative real‐time polymerase chain reaction (RT‐qPCR)

Total cellular RNA was extracted using TRIzol reagent (Invitrogen, Waltham, MA), and PCR was conducted using SYBR Premix Ex Taq (Takara, Shiga, Japan) and iCycler (Bio‐Rad, Feldkirchen, Germany), according to the manufacturers's protocol. Relative transcription levels of human dentin matrix protein 1 (DMP 1), dentin sialophosphoprotein (DSPP), bone sialoprotein (BSP), and osterix (OSX) were analysed using glyceraldehyde‐3‐phosphate dehydrogenase (GAPDH) as a normalized reference. Forward and reverse primers used are listed in Table S1.^30^, ^31^

#### 2.3.3 Western Blot assay

Seven days after osteoinduction, Western blotting was conducted as described in our previous study,^28^ using primary antibodies against DMP 1 (ab103203, Abcam), DSPP (sc‐73632, Santa Cruz Biotechnology, Dallas, TX), BSP (ab52128, Abcam), OSX (ab94744, Abcam), and VEGF (sc‐57496, Santa Cruz Biotechnology), with GAPDH as an internal control. Membranes were visualized using a chemiluminescent reagent (Sigma‐Aldrich) under a Western blotting imaging system (Bio‐Rad, Hercules, CA), and protein expression was quantified using ImageJ software.

### 2.4 Animal experiment on a semi‐orthotopic tooth root fragment model

#### 2.4.1 Preparation of root fragments of human teeth

Mature single‐root premolars without previous root canal treatment were collected from adult patients. Tooth root fragments were prepared as previously reported.^32^ Briefly, 5‐mm fragments were sectioned with pulp tissue, pre‐dentin, and partial dentin removed. Canals were enlarged to 3 mm in diameter, treated with 5% EDTA for 5 min, and subjected to ultrasonic treatment for 10 min. The larger end was then sealed with mineral trioxide aggregate. Fragments were stored in PBS containing 50 mg/ml streptomycin and 50 U/ml penicillin at 4℃ and disinfected by UV sterilization. The fluid hydrogel containing 4 × 10^5^ DPSCs from each group was injected into the canal cavity of root fragments. The hydrogel‐DPSCs compounds jelled quickly to prevent leakage.

#### 2.4.2 Subcutaneous implantation into nude mice

Animal experiments were approved by the Institutional Review Board of the Stomatological Hospital, Southern Medical University (201806) and conformed to ethical principles. Surgical procedures were performed as previously reported.^32^ Briefly, surgery was performed on 6‐week‐old immunocompromised nude mice (*n* = 12) under general anaesthesia. A single hydrogel‐DPSCs filled fragment was transplanted subcutaneously into the dorsal side of each mouse. All mice were euthanized at 4 weeks post‐surgery, and all fragments were collected.

#### 2.4.3 Histological assay

As per our previous protocol, fragments were fixed in 4% paraformaldehyde overnight, decalcified for 3 months using 10% EDTA, embedded in paraffin, sectioned into 5‐μm slices, and subjected to H&E and Masson's trichrome staining (Solarbio, Beijing, China). Histomorphology was observed using Scanscope CS and Image Scope software (Aperio, Sausalito, CA). Quantitative analysis was conducted using ImageJ software to measure the percentage of regenerated pulp tissue area inside the root canal, the percentage of pulpal blood vessel area, and the number of cells attached to the dentin wall.

#### 2.4.4 Odontogenic immunohistochemical staining

Immunohistochemistry analysis was conducted using an HRP‐DAB Cell & Tissue Staining Kit (R&D Systems, Minneapolis, MN). Briefly, samples were deparaffinized, blocked, and incubated with anti‐DMP1 (Abcam) overnight at 4℃, followed by incubation with horseradish peroxidase‐conjugated secondary antibodies for 30 min after sample washing. Histometric observations were performed as the methods in the histological assay.

### 2.5 Proteome cytokine array of CMs

Secretome analysis was conducted using a Proteome Profiler Mouse XL Cytokine Array kit ARY028 (R&D Systems) following the manufacturer's protocol. TGO‐CM and TGC‐CM at 16 h were screened for a total of 111 biofactors. Gene ontology enrichment analysis (GOEA) and Kyoto Encyclopedia of Genes and Genomes (KEGG) enrichment analysis were conducted. Data were visualized using an InnoScan 300 Microarray Scanner and analysed using GenePix Pro software and RayBiotech Q‐Analyzer software (RayBiotech, Inc., Shanghai, China).

### 2.6 Statistical analysis

Data analysis and graphical preparation were conducted using GraphPad Prism 8.3. Data are described as mean ±standard deviation and replicated for three independent experiments. One‐way ANOVA with Tukey's multiple comparison test was performed to detect statistical differences. Statistical significance was set at *p* < 0.05.

## 3 RESULTS

**FIGURE 1:**
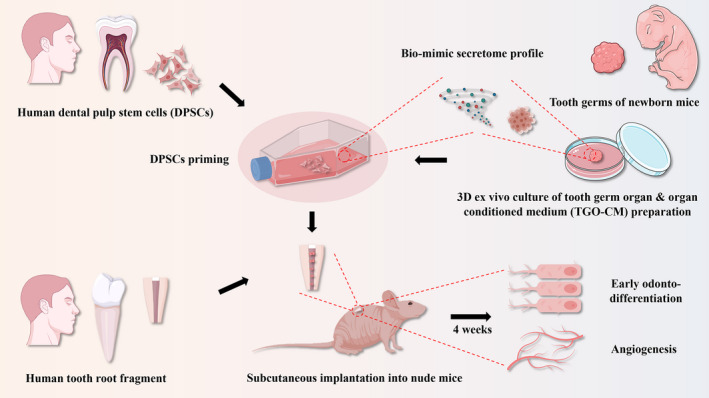
Illustration of 3D TGO‐CM primed DPSCs for early dental pulp regeneration

The study design is depicted in Figure 1.

### 3.1 Cell types within tooth germs

**FIGURE 2:**
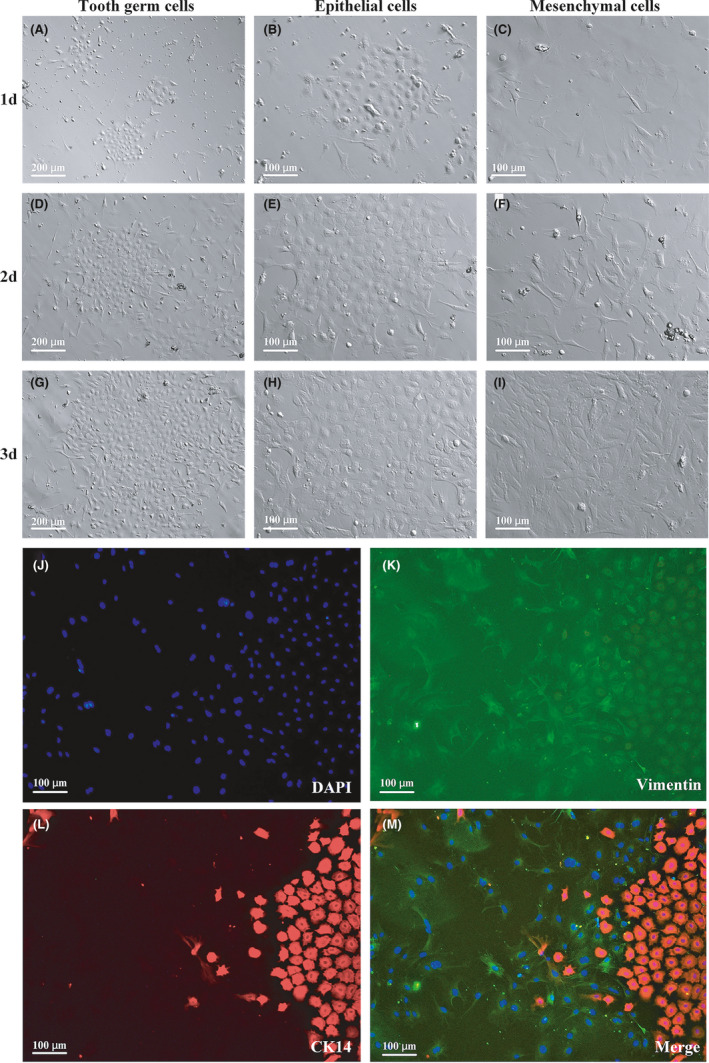
Tooth germs consisted of mesenchymal and epithelial cells. (A, D, G) tooth germ cells (TGCs) at days 1, 3, 5. TGCs were a mixture of different cell types and proliferated at a fast tempo. (B, E, H) Epithelial cells in TGCs at days 1, 3, 5 were cobblestone‐like. (C, F, I) Mesenchymal cells at day 1, 3, 5 were spindle‐like. (J–M) cobblestone‐like and spindle‐like cells were positively stained by epithelial marker CK14 and mesenchymal marker vimentin, respectively

Tooth germ cells from digested tooth germ explants (Figure 2) reached 90% confluence by day 3. Under a telescope (Figure 2A,D,G), TGCs appear to be a mixture of epithelial‐like cells, with an oval cobblestone appearance (Figure 2B,E,H), and mesenchymal‐like cells, presenting in polygonal, triangular, and spindle‐like forms (Figure 2C,F,I). Immunofluorescent staining (Figure 2J–M) showed that the epithelial marker CK14 stained the cobblestone‐like cells red, while the mesenchymal marker vimentin stained the spindle‐like cells green.

### 3.2 Viability of tooth germs under 3D ex vivo culture

**FIGURE 3:**
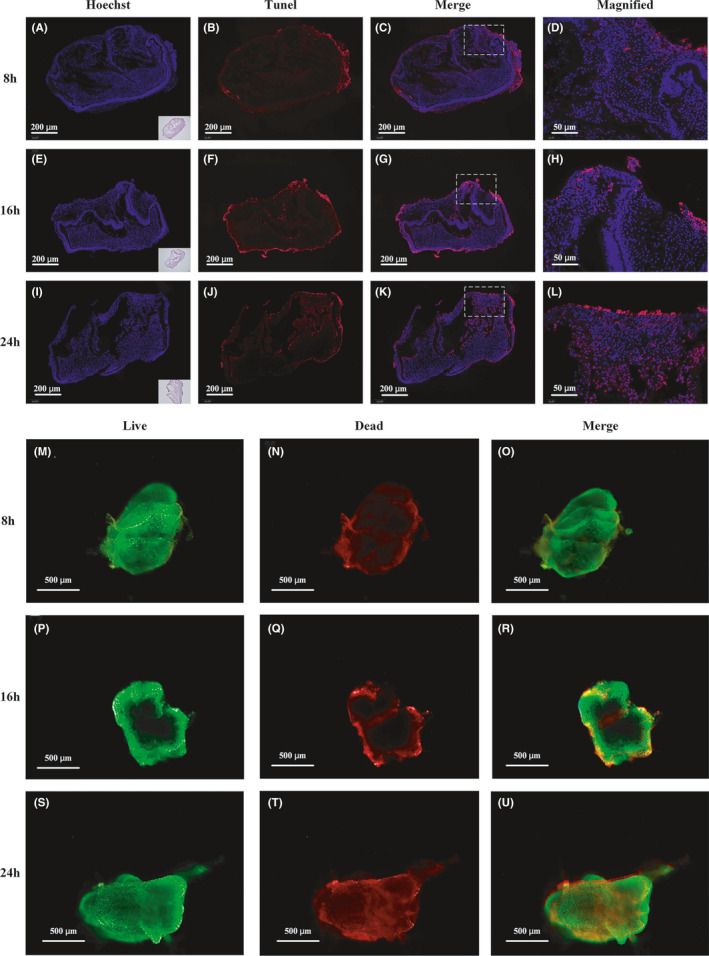
Viability of tooth germs under 3D ex vivo culture. (A–D) Cell apoptosis inside germs was undetectable at 8 h. (E–H) Slight cell apoptosis emerged at 16 h. (I–L) Grave cell apoptosis inside germs appeared at 24 h. (M–O) Cell necrosis inside germs was undetectable at 8 h. (P–R) Slight cell necrosis emerged at the centre of germs at 16 h. (S–U) Grave cell necrosis appeared at the centre of germs at 24 h. White dotted squares: (D, H, L) represented the magnified regions of (C, G, K), respectively

The Hoechst/TUNEL staining indicated that cell apoptosis within tooth germ was undetectable at 8 h, emerging at 16 h, and peaking at 24 h (Figure 3A–L). Similarly, at 8 h, the red damaged parts were barely seen inside the germ except for on the nonspecific margins; at 16 h, slight tissue necrosis (red) was observed at the centre, with the vast outer layer stained in green, indicating living tissue; at 24 h, severe tissue damage was indicated by an enlarged red core (Figure 3M–U).

### 3.3 TGO‐CM enhanced in vitro DPSCs behaviour

**FIGURE 4:**
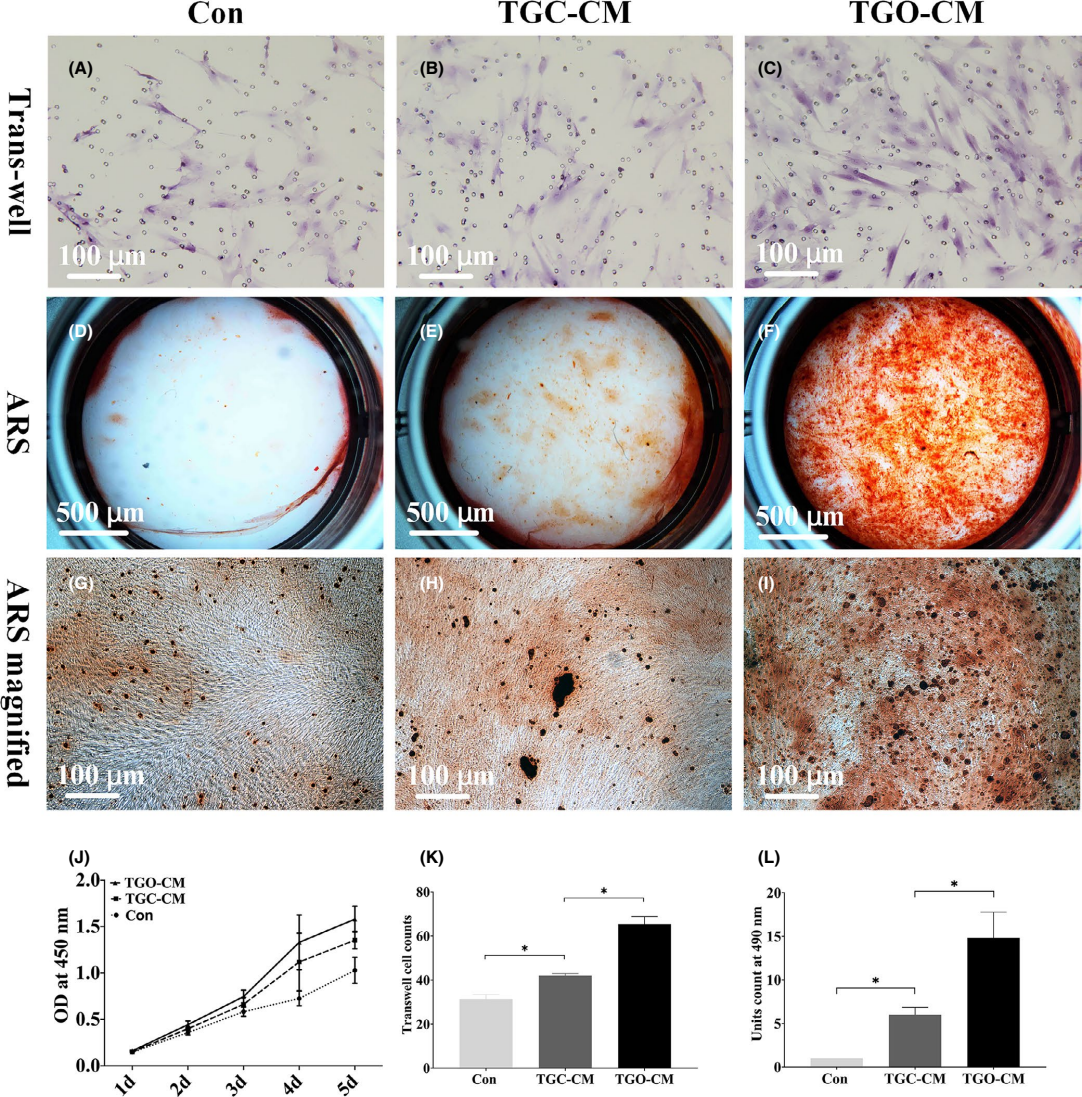
CM of 3D ex vivo cultured tooth germ organs enhanced in vitro DPSCs proliferation, migration, and in vitro mineralization. (A–C) Migrated DPSCs at 24 h primed by control medium, TGC‐CM, and TGO‐CM, respectively. (D–I) Alizarin Red S staining of DPSCs primed by control medium, TGC‐CM, and TGO‐CM for 3 weeks, respectively. (J–L) Quantitative data of DPSCs proliferation, migration, and ARS mineralized units, respectively. * indicated *p* < .05

Transwell migration assay revealed that the TGO‐CM group presented the most migrated cells (Figure 4A–C). Similarly, Alizarin Red S staining revealed a higher level of mineralized deposition in the TGO‐CM group (Figure 4D–I).

Quantitatively, DPSCs primed by TGO‐CM proliferated more quickly and had increased by 0.56 ± 0.28 and 0.22 ± 0.14 folds, respectively, compared to the control group and TGC‐CM group at day 5 (*p* < 0.05, Figure 4J). The number of migrated DPSCs in the TGO‐CM group was 1.09 ± 0.19 and 0.55 ± 0.12 folds greater than those of the control group and TGC‐CM group, respectively (*p* < 0.0001, Figure 4K). The ARS unit count in the TGO‐CM group was significantly larger, by 13.81 ± 2.96 and 8.81 ± 2.48 folds, compared to those of the control group and TGC‐CM group, respectively (*p* < 0.01, Figure 4L).

### 3.4 CM gene expression and protein levels

**FIGURE 5:**
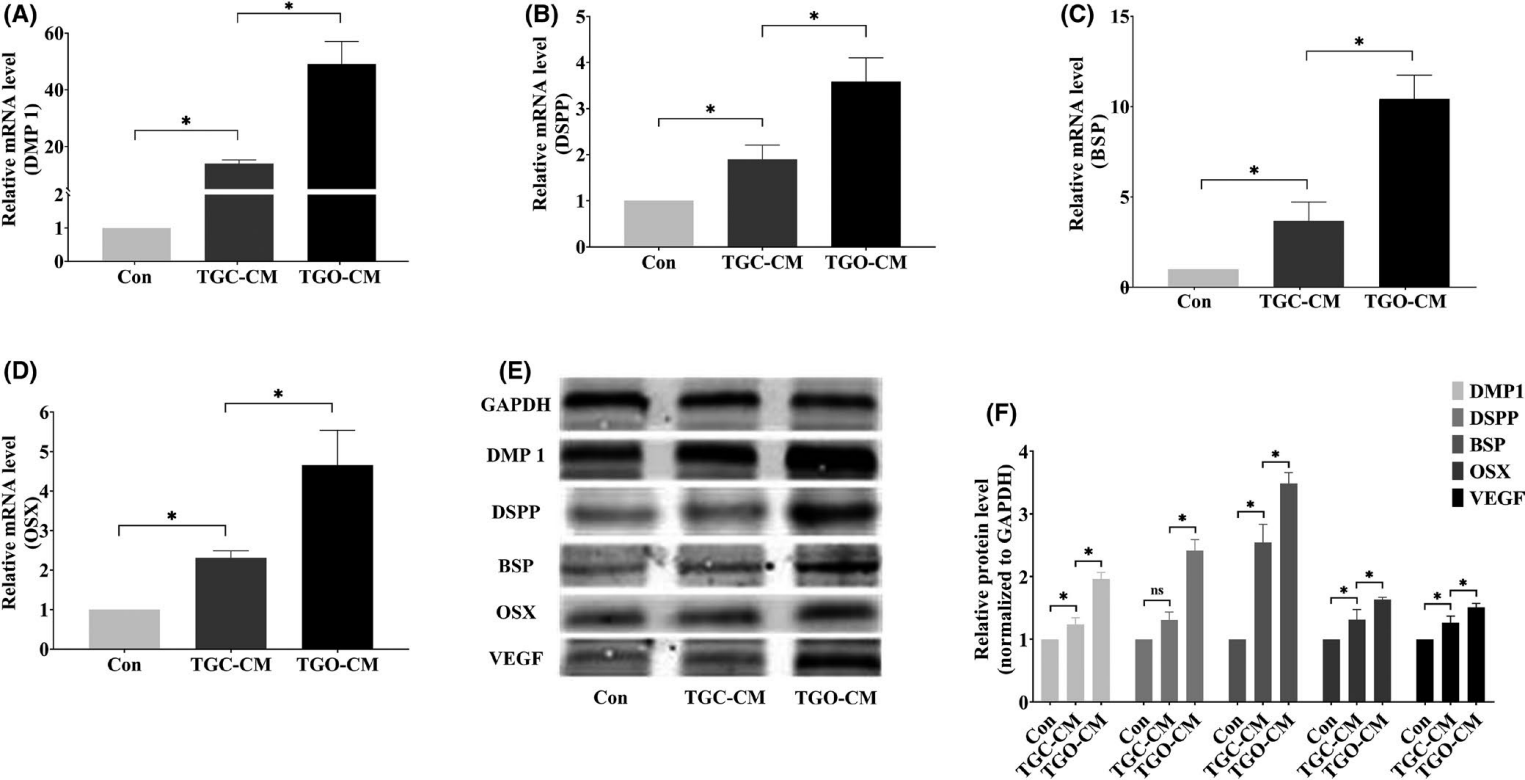
Odontogenic and angiogenic differentiation. (A–D) Expression of DMP 1, DSPP, BSP, OSX genes was elevated at mRNA level in the TGO‐CM group. (E–F) Expression of DMP 1, DSPP, BSP, OSX, VEGF was elevated at the protein level in the TGO‐CM group. * indicated *p* < .05, and “ns” suggested non‐significant

Odontogenesis‐related gene expression in the TGO‐CM group was notably elevated at the mRNA transcription level (Figure 5A–D). The relative gene expressions of the TGO‐CM group compared with the control and TGC‐CM groups were as follows, each, respectively: DMP 1, 49.10 ± 7.97 and 3.48 ± 0.27 folds greater; DSPP, 3.59 ± 0.51 and 1.89 ± 0.07 folds greater; BSP, 10.43 ± 1.32 and 3.04 ± 1.06 folds greater; and OSX, 4.66 ± 0.89 and 2.02 ± 0.40 folds greater (*p* < 0.01).

Odontogenic and angiogenic protein levels were elevated in the TGO‐CM group (Figure 5E). The relative protein levels of the TGO‐CM group compared with the control and TGC‐CM groups were as follows, each, respectively: DMP 1, 1.96 ± 0.10 and 1.59 ± 0.11 folds greater; DSPP, 2.42 ± 0.17 and 1.86 ± 0.20 folds greater; BSP, 3.49 ± 0.17 and 1.38 ± 0.14 folds greater; OSX, 1.64 ± 0.03 and 1.26 ± 0.13 folds greater; and VEGF, 1.51 ± 0.06 and 1.20 ± 0.11 folds greater (*p* < 0.05, Figure 5F).

### 3.5 In vivo dental pulp regeneration

**FIGURE 6:**
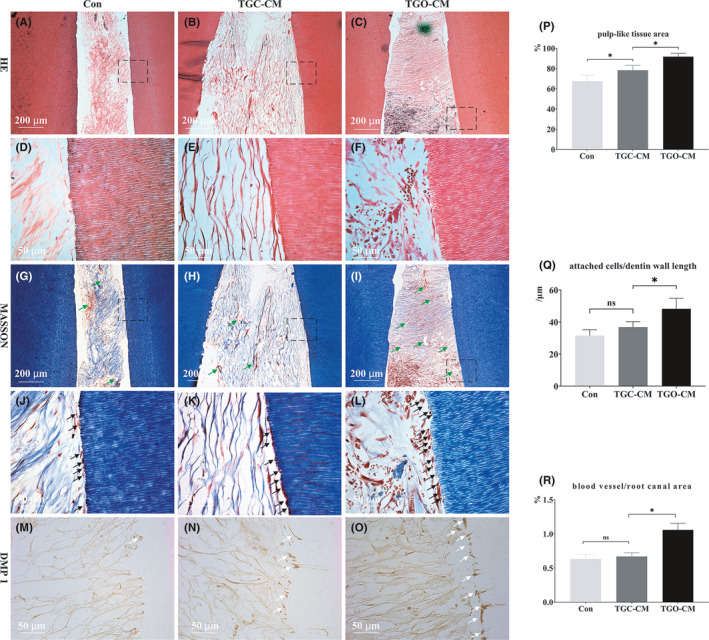
CM of 3D ex vivo cultured tooth germ organs promoted in vivo pulp regeneration. (A, D, G, J) and (B, E, H, K) Poorly organized and hypo‐vascular connective tissue healed in the control group and TGC‐CM group, respectively. (C, F, I, L) Well‐organized and vascularized pulp tissue regenerated in the TGO‐CM group. (M–O) The attached cell layer in the TGO‐CM group was deeply stained with DMP 1, indicating the odontogenic odontoblast‐like cells. (P–R) 3D TGO‐CM increased the area of regenerated pulp‐like tissue and the number of attached cells and blood vessels. Black dotted squares: (D, E, F) and (J, K, L) represented the magnified regions in (A, B, C) and (G, H, I), respectively. Green arrows: blood vessels. * indicated *p* < .05, and “ns” suggested non‐significant

Within the 4 weeks, all surgical sites healed without observable inflammation, infection, or graft exposure. The grafted hydrogel was fully degraded in all groups (Figure 6). Root canals in the control group were filled with fibre‐rich tissue containing a trivial number of cells. The resulting mass of poorly organized, deformed, hypo‐vascular connective tissue bore no similarities to pulp tissue (Figure 6A,D,G,J). The TGC‐CM group demonstrated increased cells and capillary vessels but without organized pulp tissue structure (Figure 6B,E,H,K). As depicted in Fig. C and I, canals of the TGO‐CM group were almost filled with compacted connective tissue. The pulp‐like tissue was well structured with ECM, collagen, abundant cells, and a robust blood vessel network (green arrows, Figure 6C,F,I,L). Higher Masson magnifications (Figure 6J–L) revealed an uneven cell layer situated at the dentin–pulp interface, attached to the dentin wall, in both the TGM‐CM and TGO‐CM groups. However, cells in the former displayed a flat appearance, while those of the latter appeared more columnar. Immunohistochemical staining of DMP 1, an odontogenic marker, revealed deeper staining in the attached cell layer in the TGO‐CM group, identifying odontogenic odontoblast‐like cells (white arrows, Figure 6M–O). However, no visible mineralized dentin‐like tissue formation or detectable odontoblast polarity was detected in all groups.

Quantitatively, the characteristics of the TGO‐CM group in comparison with the control and TGC‐CM groups were as follows, each, respectively: pulp‐like tissue filling rate, 0.92 ± 0.03 in the TGO‐CM, and 0.37 ± 0.11 and 0.18 ± 0.12 folds greater (*p* < 0.01); number of attached cells, 0.54 ± 0.21 and 0.31 ± 0.13 folds greater (*p* < 0.05); blood vessel area rate, 0.69 ± 0.30 and 0.60 ± 0.27 folds greater (*p* < 0.01) (Figure 6P–R).

### 3.6 Protein profile assay of CMs

**FIGURE 7:**
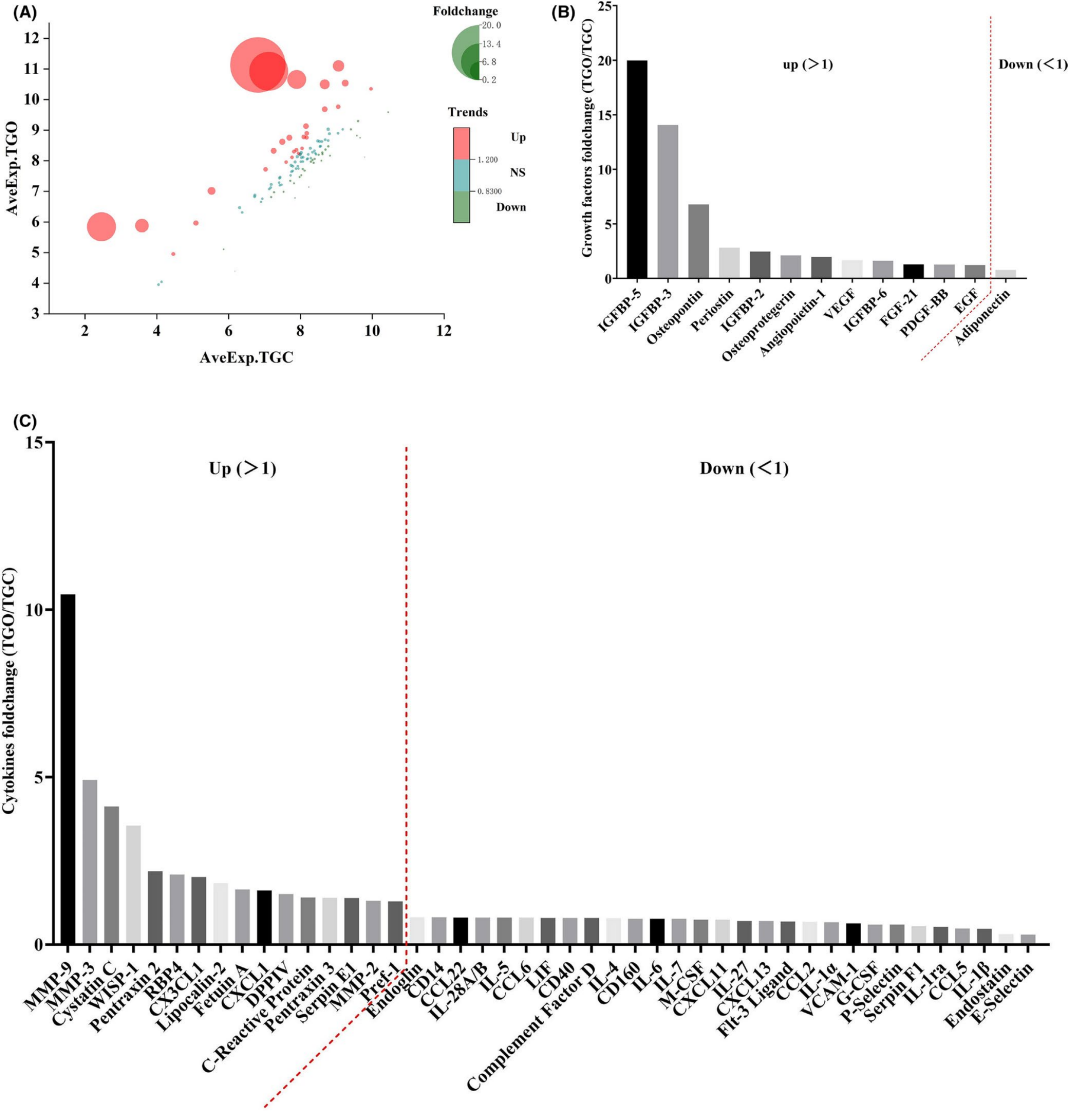
Protein profile differences between TGO‐CM and TGC‐CM. (A) Fifty‐eight types of differential proteins were detected with statistical significance. (B) TGO‐CM contained higher levels of growth factor types. (C) TGO‐CM contained fewer pro‐inflammatory cytokines

Of the 111 proteins screened, 58 were detected with statistical significance in both CM types (Figure 7A). TGO‐CM contained higher levels of insulin‐like growth factor‐binding protein 3 and 5 (IGFBP 3 and 5, 14.05‐ and 19.99 folds, respectively), osteopontin (6.79 folds), periostin (2.82 folds), osteoprotegerin (2.11 folds), angiopoietin‐1 (1.97 folds), VEGF (1.66 folds), fibroblast growth factor (FGF, 1.28 folds), platelet‐derived growth factor (PDGF, 1.27 folds), and epidermal growth factor (EGF, 1.23 folds). TGC‐CM contained more adiponectin (Figure 7B). In addition, TGO‐CM contained higher levels of angiogenesis‐related cytokines like matrix metallopeptidase 9 (MMP‐9, 10.45 folds), serpin E1 (1.39 folds), and CX3CL‐1 (2.02 folds), and contained fewer pro‐inflammatory factors such as interleukin‐1 α and β (IL‐1 α and β, 0.67‐ and 0.47 folds, respectively), interleukin‐6 (IL‐6, 0.77 folds), macrophage colony‐stimulating factor (M‐CSF, 0.74 folds), and granulocyte colony‐stimulating factor (G‐CSF, 0.60 folds) (Figure 7C).

### 3.7 Possible mechanisms leading to the protein profile differences

**FIGURE 8:**
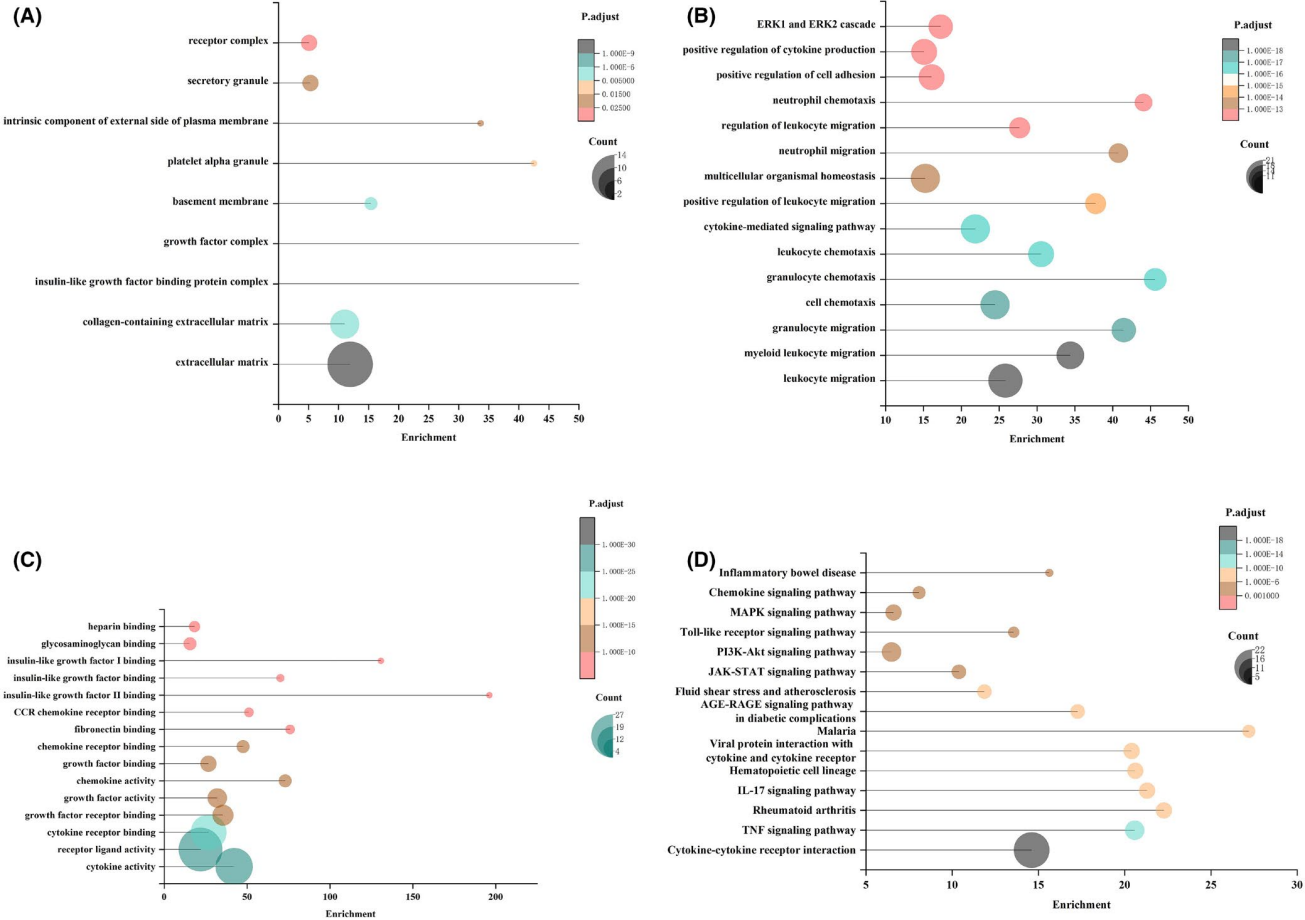
Possible mechanisms that led to the protein profile differences. (A) GO cellular component analysis: differential proteins mainly existed in forms of the receptor complex, secretory granule, growth factor complex, and extracellular matrix. (B) GO biological process analysis: differential proteins were mainly linked to cytokine production, positive cell adhesion regulation, and inflammatory processes. (C) GO molecular function analysis: differential proteins were functionally related to the activity and receptor bindings of growth factors, chemokines, and cytokines (D) KEGG analysis: cytokine–cytokine receptor interaction, TNF signalling pathway, and PI3K‐Akt signalling pathway may be involved

GO cellular component analysis (Figure 8A) revealed that the differential proteins existed in forms of the receptor complex, secretory granule, growth factor complex, and extracellular matrix, with 5, 5, 6, and 23 detected protein types, respectively (*p* < 0.05). GO biological process analysis confirmed that these differential proteins were linked to cytokine production, positive cell adhesion regulation, and inflammatory processes (Figure 8B). These results aligned with the downregulated inflammation‐related cytokine activity in TGO‐CM (Figure 7C, *p* < 0.05). GO molecular function analysis (Figure 8C) revealed that these proteins were functionally related to the activity and receptor bindings of growth factors, chemokines, and cytokines (*p* < 0.05). KEGG analysis identified possible signalling mechanisms that led to such differential protein profile, including cytokine–cytokine receptor interaction, TNF signalling pathway, and PI3K‐Akt signalling pathway (Figure 8D).

## 4 DISCUSSION

Tooth germ comprises mesenchymal and epithelial components, and its organogenesis is a temporal interplay involving diverse biofactors between these two elements.^25^, ^33^ Successful CM production requires careful collection timing; 2D CM can be harvested between 24–72 h and every 2–4 days after seeding.^19^ 3D ex vivo culture of tooth germs mimics the physiology of the original microenvironment, with hierarchical tissue structures and graded chemical distribution.^23^, ^24^ In our study of the 3D culture, hypoxia, undernutrition, and accumulation of metabolic waste resulted in greater damage at the organ core. The elevated apoptosis rate in tissue culture compared to that in cell line culture can distort the protein secretion profile^34^; excessive tissue debris and by‐products may interfere with the subsequent CM content analysis.^35^ Thus, to counter the tissue damage presented at 24 h and the low protein secretion at 8 h, 16 h was adopted as the harvest timing.

Vectors‐mediated MSCs engineering has also been reported to successfully prime stem cells.^36^, ^37^ However, higher technique sensitivity, higher cost, and longer lab training curve have all limited its broad application from a clinical perspective. Besides, MSCs derived from parental pluripotent stem cells (PSC‐MSCs) have shown advantages over adult MSCs such as higher homogeneity and proliferative potency.^38^, ^39^ Nevertheless, the human ethical concerns, cell availability, and subsequent cell expansion process all limited their application. Thus, in this study, we chose the TGC‐CM and TGO‐CM, instead of the direct engineered MSCs or PSC‐MSCs, to prime DPSCs for pulp repair.

Compared with the 2D TGC‐CM, the 3D TGO‐CM demonstrated a superior DPSCs priming capability with improved cell proliferation, migration, in vitro mineralization, odontogenic differentiation, and angiogenesis. Conventional 2D CM‐based revitalization therapy fails to regenerate truly functional dental pulp.^40^ Odontoblast‐like cells and vasculature are two intrinsic features of dentin–pulp complex regeneration. Non‐collagenous proteins secreted by odontoblasts include odontogenesis‐related DMP 1, DSP, BSP, and osteogenesis‐related OSX.^41^ VEGF plays a pivotal role in angiogenesis by promoting endothelial cell survival, proliferation, and migration.^42^ Vasculature networks are essential in nutrient and cytokine delivery and metabolic waste transportation during pulp repair.^43^

Semi‐orthotopic models using subcutaneous tooth fragment implantation have been broadly adopted to investigate pulp regeneration,^32^, ^44^, ^45^, ^46^ and such studies using 2D CM have demonstrated positive results of pulp–dentin complex regeneration. Here, we present the first semi‐orthotopic model‐based study to investigate DPSCs priming by 3D CM and DPSCs‐mediated pulp repair. The formation of a well‐organized pulp structure, robust vascular network, and odontogenic cell layer in the 3D TGO‐CM group proved its superior regenerative capability over that of the 2D TGC‐CM. However, no pre‐dentin formation and odontoblast polarity were observed, which may be ascribed to the relatively short 4 weeks in vivo experimental time. Similar studies typically euthanize subjects after 12 weeks, providing sufficient time for hard tissue formation and odontoblast polarization.^32^, ^44^, ^45^, ^46^ We anticipate a proportionate trajectory in increased hard tissue formation and odontoblast polarization to accompany a longer timeline. Interestingly, the DMP1‐positive cells were morphologically distinct, with flat and columnar structures in the 2D TGC‐CM and 3D TGO‐CM groups, respectively, indicating a polarization trend. The origin of the regenerated pulpal vasculature in this study remains unclear. Vasculature can be derived either from angiogenesis via host vessel growth, or from vasculogenesis via angiogenic DPSCs differentiation.^47^ Here, lacking labelling analysis, we speculated that the neovascularization was angiogenesis which was derived from host vessel ingrowth through the end opening.^48^ Summarily, in vitro and in vivo evidence confirmed the potential of 3D TGO‐CM as a priming cocktail to enhance DPSCs‐mediated pulp regeneration.

Proteome cytokine array, GOEA, and KEGG analysis revealed significant differences between the two CM secretome profiles. TGO‐CM contains more growth factors such as FGF and EGF, which are related to cell proliferation and odontogenic differentiation.^2^ Higher levels of angiogenesis‐related factors such as angiopoietin‐1, PDGF, VEGF, and FGF, in TGO‐CM, contributed to vasculature formation.^49^ Higher level of CX3CL‐1, a type of chemokine with an angiogenic role,^50^ was also detected in TGO‐CM. In a clinical scenario, pulp blood perfusion is often limited by a narrow apical foramen or progressive inflammation,^51^ potentially causing hypoxia in implanted DPSCs. 3D ex vivo culture of tooth germ mimics the in vivo graded oxygen distribution during germ development, with its inner core exhibiting increased hypoxia. As a self‐protective response, the tooth germ elevates related gene expression, secreting more biofactors associated with cell survival, proliferation, and angiogenesis. Our results correspond with these observations, demonstrating that hypoxia can guide MSCs towards a pro‐angiogenic phenotype and promote angiogenesis.^52^, ^53^, ^54^

Inflammation plays an important role in pulp regeneration,^55^ as tissue repair can only occur at a lower inflammation resolution level.^56^ An inflammatory biomolecules influx would impede regeneration due to cellular apoptosis and tissue necrosis^57^, ^58^; however, low‐intensity inflammation would stimulate positive pulp reparative responses.^59^, ^60^ Typical pro‐inflammatory cytokines that govern the pulp inflammation process include interleukins like IL‐1α/β and IL‐6, collagenases like MMPs, colony‐stimulating factors like M‐CSF and G‐CSF, and chemokines like CXCLs and CCLs.^61^ MMP‐3 promotes pulp regeneration in mild irreversible pulpitis by inhibiting IL‐6 activity.^62^ Wnt/β‐catenin pathway activation in the noncanonical Wnt/Ca2+ pathway is implicated in impairing DPSC‐based odontogenesis under inflammatory conditions.^63^ Mild inflammatory conditions facilitate pulp repair through intracellular p38 MAPK and NF‐κB signalling pathways.^59^, ^60^ Moreover, the paracrine function of MSCs could be strictly regulated by inflammatory signalling pathways like critical telomerase associated protein RAP 1/NF‐kB signalling pathway.^64^, ^65^ Future efforts are still required to further elucidate the inflammation modulation and immunomodulation of MSCs. Here, in this study, the 3D TGO‐CM created a mild inflammatory cocktail with less pro‐inflammatory cytokines than the 2D TGC‐CM.

The GO biological process analysis paralleled those of the molecular function analysis. Growth factors, cytokines, and chemokines are all present in TGO‐CM. Though not entirely conclusive, the KEGG results imply that the differences between the secretome profiles may be attributable to the cytokine–cytokine receptor interaction and PI3K‐Akt signalling pathway. Cytokines bind to receptors on the cell membrane surface, activate intracellular biomolecule interactions, and subsequently induce various cell behaviour, such as inflammatory responses. The PI3K‐Akt signalling pathway plays a major role in cell apoptosis mitigation, proliferation, differentiation, and angiogenesis.^66^ Growth factors bind to protein G‐linked receptors, activate P13K/AKT, and induce subsequent cell activities. Hypoxia promotes angiogenesis via the interaction between hypoxia‐inducible factor‐1α (HIF‐1α) and the P13K/AKT signalling pathway.^67^ In this study, the biomimic‐graded hypoxia inside 3D cultured tooth germs may induce the production of pro‐angiogenic biomolecules as an adaptive response.

Due to budget and time constraints, we were unable to address certain limitations. Future efforts may focus on the following aspects: influence of factors such as different embryonic stages of tooth germs, priming duration, and pathological status on protein profiles; more thorough filtering of effective CM components; in‐depth mechanisms leading to different secretome profiles; use of orthotopic models of larger animals and longer‐term follow‐up; optimizing and standardizing the fabrication protocols of 3D CM before use.

## 5 CONCLUSION

Dental pulp stem cells‐based pulp regeneration exhibit shortcomings, and its therapeutic outcomes rely heavily on the unreliable post‐implantation functional status of stem cells. In this study, we prepared CM of 3D tooth germ organ and investigated its preconditioning capabilities on DPSCs for early dental pulp regeneration on a semi‐orthotopic tooth fragment model in nude mice. 3D TGO‐CM primed DPSCs exhibited higher cell proliferation, migration, in vitro mineralization, odontogenic differentiation, and angiogenesis. Moreover, 3D TGO‐CM priming achieved superior early in vivo pulp regeneration results, with well‐organized pulp structures, odontogenic cell layer attachment, and increased vasculature formation at 4 weeks post‐surgery. 3D TGO‐CM contained more growth factors related to odontogenesis and angiogenesis and fewer pro‐inflammatory cytokines. Cytokine–cytokine receptor interaction and PI3K‐Akt signalling pathway may account for the differential protein profiles of the two CM types.

To the best of our knowledge, this is the first study to investigate the protein profile of 3D tooth germs and the first to apply it for early in vivo pulp regeneration. Taken together, our results confirmed that 3D TGO‐CM can be applied as a priming cocktail to enhance DPSCs‐mediated early pulp regeneration. This study may shed light on personalized stem cell priming for early pulp regeneration using a trace‐back‐to‐organ approach.

## CONFLICT OF INTEREST

There are no conflicts of interest in this study.

## AUTHOR CONTRIBUTION

Zhihui Tian and Mingdeng Rong were involved in study design; Zijie Wang, Tengfei Zhou, and Zhihui Tian helped in experiment conduction; Hongxing Chu, Chuying Chen, and Jiayi Zhang contributed to data collection & analysis; Tengfei Zhou was involved in manuscript drafting; Mingdeng Rong and Zhihui Tian helped in manuscript review. This study is supported by Scientific Research Talent Cultivation Project of Stomatological Hospital, Southern Medical University (RC202007), President Foundation of Nanfang Hospital, Southern Medical University (2018B014), and Natural Science Foundation of Guangdong Province, China (2021A1515011656).

## Supporting information

Tab S1

## Data Availability

Data are available from the corresponding author upon reasonable request.
