# Supplementary material for: Conditioned medium derived from 3D tooth germs: A novel cocktail for stem cell priming and early in vivo pulp regeneration
Source: Cell Prolif. 2021 Sep 28;54(11):e13129. doi: 10.1111/cpr.13129 (PMC8560607; doi:10.1111/cpr.13129)
Supplement: Supplementary file 1 — Tab S1 [file CPR-54-e13129-s001.docx]

**Conditioned medium derived from 3D tooth germs: a novel cocktail for stem cell priming and early in vivo pulp regeneration**

**Corresponding author**

**Name:** Zhihui Tian

**Email:** Tianzh@i.smu.edu.cn

**Affiliation:**

1. Department of Stomatology, Nanfang Hospital, Southern Medical University, Guangzhou, 510515, China.

2. School of Stomatology, Southern Medical University, Guangzhou, 510515, China.

**Phone number:** +86 13602770886

**Fax number:** +86 020 62787679

**Table S1 primer sequences for RT-qPCR**

| **Genes** | **Forward primer** | **Reverse primer** |
| --- | --- | --- |
| *GAPDH* | GGAGCGAGATCCCTCCAAAAT | GGCTGTTGTCATACTTCTCATGG |
| *DMP 1* | TTCCTCTTTGAGAACATCAACCTG | ACTCACTGCTCTCCAAGGGT |
| *DSPP* | AAAGTGGTGTCCTGGTGCAT | CCTGGATGCCATTTGCTGTG |
| *BSP* | CACTGGAGCCAATGCAGAAGA | TGGTGGGGTTGTAGGTTCAAA |
| *OSX* | TCTGCGGGACTCAACAACTC | TAGCATAGCCTGAGGTGGGT |
